# CXC and CC Chemokines as Angiogenic Modulators in Nonhaematological Tumors

**DOI:** 10.1155/2014/768758

**Published:** 2014-05-29

**Authors:** Matteo Santoni, Sergio Bracarda, Massimo Nabissi, Francesco Massari, Alessandro Conti, Emilio Bria, Giampaolo Tortora, Giorgio Santoni, Stefano Cascinu

**Affiliations:** ^1^Clinica di Oncologia Medica, AOU “Ospedali Riuniti ,” Università Politecnica delle Marche, Via Conca, 60020 Ancona, Italy; ^2^UOC Medical Oncology, Department of Oncology, AUSL8, 52100 Arezzo, Italy; ^3^School of Pharmacy, Section of Experimental Medicine, University of Camerino, 62032 Camerino, Italy; ^4^Medical Oncology, Azienda Ospedaliera Universitaria Integrata, University of Verona, Piazzale L.A. Scuro 10, 37124 Verona, Italy; ^5^Dipartimento di Scienze Cliniche Specialistiche ed Odontostomatologiche, Clinica di Urologia, AOU Ospedali Riuniti, Università Politecnica delle Marche, Via Conca 71, 60126 Ancona, Italy

## Abstract

Chemokines are a superfamily of structurally homologous heparin-binding proteins that includes potent inducers and inhibitors of angiogenesis. The imbalance between angiogenic and angiostatic chemokine activities can lead to abnormalities, such as chronic inflammation, dysplastic transformation, and even tumor development and spreading. In this review, we summarize the current literature regarding the role of chemokines as modulators of tumor angiogenesis and their potential role as therapeutic targets in patients with nonhaematological tumors.

## 1. Introduction

Angiogenesis means the growth of new blood vessels from preexisting vessels and is essential to several physiologic conditions that include embryonic development, wound repair, and the ovarian/menstrual cycle. In contrast, aberrant or pathological angiogenesis is highly associated with chronic inflammation, fibroproliferative disorders, tumor growth, and progression [1–3].

In normal tissues, the balance of proangiogenic and antiangiogenic growth factors and proteins is believed to be tilted towards antiangiogenesis because of the high metabolic cost of this process. During wound repair, the formation of granulation tissue is associated with a rapidly increasing rate of normal capillary endothelial cell turnover. The first step requires the production of proteases to degrade the basement membrane and the invasion of the surrounding extracellular matrix by proliferating and migrating endothelial cells [4, 5]; thereafter, they will organize into functioning capillaries invested by a new basal lamina [6, 7]. In addition to local mechanisms, the recruitment of circulating progenitor endothelial cells to areas of angiogenesis contributes to this process [8].

In contrast, the latter phases of wound repair are associated with the onset of angiostasis and the attenuation of the angiogenic signals [9]. Mechanisms of* in vivo* angiostasis remain to be fully elucidated but may include the induction of endothelial cell apoptosis [10, 11] and the inhibition of the recruitment of angiogenic factor-producing leukocytes [12].

Inflammation and angiogenesis, while being distinct and separable processes, are closely related events because of the ability of both endothelial cells and leukocytes to respond to common stimuli, such as chemokines [13]. Chemokines are a superfamily of structurally homologous heparin-binding cytokine molecules that can promote immune responses and stem-cell survival, as well as triggering chemotaxis and angiogenesis [14]. Structurally, chemokines are grouped into 4 families (designated CC, CXC, C, and CX3C), depending on the spacing or presence of four conserved cysteine residues near their amino-terminus. In the CC subgroup, the first two cysteine residues are adjacent, whereas in the CXC subgroup the first 2 cysteine residues are separated by a nonconserved amino acid residue (hence the CXC designation). The CXC chemokine ligands are further classified on the basis of the presence or absence of three amino acid residues (Glu-Leu-Arg; “ELR” motif), preceding the first conserved cysteine amino acid residue in the primary structure of these proteins [15–17]. The CXC chemokines with the “ELR” motif (ELR+ or ELR_1_) and several of the CC chemokines are potent promoters of angiogenesis, whereas members that are induced by interferons and lack the ELR motif (ELR− or ELR_2_) are potent angiogenic inhibitors [17]. Additionally, some chemokines might also act as organ-specific angiogenesis modulators, accordingly with emerging observations on the existence of organ-specific molecules regulating vessel formation [18]. ELR+ CXC chemokines play a crucial role in the tumor growth of a variety of solid tumors, including lung, colorectal, pancreatic, ovarian, prostate, melanoma, brain, and renal cell cancer (Table 1) [19].

This review will discuss the current literature regarding the role of chemokines as modulators of angiogenic or angiostatic responses.

## 2. CXC Chemokines, CXC Chemokine, CXC Chemokine Receptors, and Angiogenesis

The members of the CXC family are among the first chemokines identified as regulators of angiogenesis, acting in a disparate manner in the promotion or inhibition of angiogenesis [46]. This family includes CXC ligand 1 (CXCL1) (growth-related oncogene-a (GRO-a)), CXCL2 (GRO-b), CXCL3 (GRO-g), CXCL4 (platelet factor-4 (PF-4)), CXCL5 (epithelial neutrophil-activating peptide-78 (ENA-78)), CXCL6 (granulocyte chemotactic protein-2 (GCP-2)), CXCL7 (neutrophil-activating peptide-2 (NAP-2)), CXCL8 (interleukin-8 (IL-8)), CXCL9 (monokine induced by interferon-g (IFN-*γ*) (MIG)), CXCL10 (IFN-*γ*-inducible protein-10 (IP-10)), CXCL11 (IFN-inducible T-cell a chemoattractant (I-TAC)), CXCL12/SDF-1 (stromal derived factor-1*α* (SDF-1*α*)), CXCL13 (B-cell chemoattractant-1 (BCA-1)), CXCL14 (breast and kidney-expressed chemokine (BRAK)), and CXCL16 [14, 15].

The CXC chemokines have been shown to interact with the CXC chemokine receptor (CXCR) family of molecules. To date, five CXC receptors (CXCR1–5) have been identified in various human cell lines. They are members of the rhodopsin-like seven-transmembrane G protein-coupled receptor family [47–49]. CXCR1, which is also known as IL-8RA, binds to CXCL6 and CXCL8 with high affinity [49]. CXCR2 has been shown to bind to CXCL1, CXCL2, CXCL3, CXCL5, CXCL6, CXCL7, and CXCL8 [49, 50], acting as the common mediator for their angiogenic activity, whereas CXCR3 has been shown to bind to CXCL9, CXCL10, CXCL11, CXCL4, and CXCL4L1 [47].

CXCR4 has been shown to bind to CXCL12/SDF-1, which belongs to a subgroup of constitutively expressed chemokines involved in the maintenance of leukocyte trafficking during homeostasis. The fundamental role of CXCR4 in the formation of gastrointestinal tract arteries, as well as in vessel development, hematopoiesis, and cardiogenesis, has been demonstrated in studies in CXCR4 and CXCL12/SDF-1 gene-deficient mice [51]. Thus, CXCL12/SDF-1 upregulates VEGF-A production and VEGF-A upregulates CXCR4 expression, thus generating an amplification circuit crucially influenced by hypoxia [52]. CXCR4 antagonists include low-molecular-weight molecules, such as Plerixafor (AMD3100) [53] and MSX-122 [54], and peptides, such as ALX40-4C or the polyphemusin analogues (TN14003/BKT140), T22, and CTCE-9908 (Table 2).

As regards to CXCR5, it is found mostly on B cells and is responsible for B cell chemotaxis mediated by B cell-attracting chemokine 1 and BCR-triggered B-cell activation [55, 56].

The list of CXCRs includes a sixth member, DARC or the Duffy Ag receptor for chemokines [57, 58], which binds to CXCL8, CXCL1, and CXCL7, although this receptor can also bind to various CC chemokines, such as RANTES and monocyte chemotactic protein-1 [59]. Notably, another receptor is encoded by an open reading frame from* Herpesvirus saimiri* and has been shown to bind to ELR+ CXC chemokines [60].

## 3. Role of ELR+ CXC Chemokine and CXCL12/SDF-1 ***α*** in Promoting Tumor Angiogenesis

**Figure 1:**
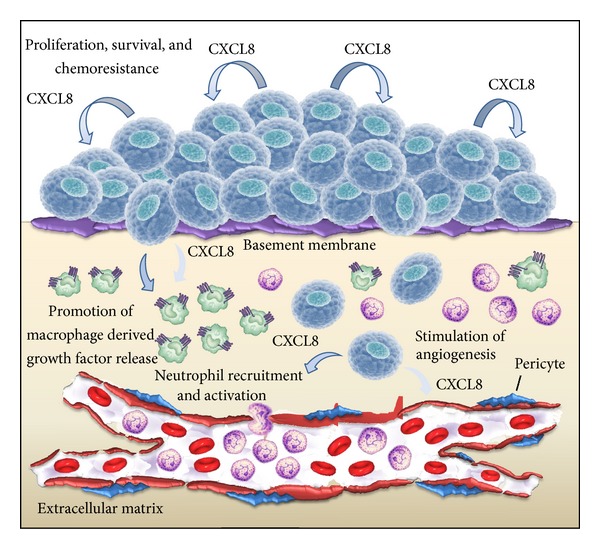
The role of CXCL8 signaling in the tumor microenvironment. Autocrine CXCL8 production by cancer cells can enhance their proliferation and survival of cancer cells through autocrine signaling pathways. Tumor-derived CXCL8 promotes angiogenesis, cell invasion, and migration. In addition, CXCL8 induces a chemotactic infiltration of neutrophils into the tumor site and the secretion of additional growth factors by tumor-associated macrophages.

The establishment of a proangiogenic tumor environment is the result of an uncontrolled overexpression of angiogenic factors or an inappropriate suppression of angiostatic molecules. This imbalance promotes tumor growth, survival, invasion, and metastases. ELR+ CXC chemokines, such as CXCL-8 (Figure 1), have been determined to play a critical role in tumor growth and metastases. This process is mediated by several mechanisms, including the activation of seven transmembrane G protein coupled receptors (7TM-GPCR) and protein tyrosine kinase receptors (PTKR). They contribute to the expression of angiogenic CXC chemokines via NF-*κ*B activation in cancer cells, thus enhancing tumor-associated angiogenesis [61, 62] and suggesting for a potential role of 7TM-GPCRs, such as CXCR2, and PTKR in preneoplastic to neoplastic transformation. This notion is supported by the evidence that, in a model of syngeneic renal cell carcinoma, CXCR2 ligand expression increased with tumor growth, whereas there was a significant inhibition in the CXCR2^−/−^ mice that correlated with decreased angiogenesis and necrosis. Otherwise, in CXCR2^−/−^ mice, the orthotopic tumors had a decreased potential to metastasize to the lung [38].

The straight connection between NF-*κ*B and angiogenic CXC chemokines has been further demonstrated by Jian et al. [63]. They transfected glioblastoma cells with mutant I*κ*B*α* (I*κ*B*α*M), an inhibitory protein that sequesters and blocks NF-*κ*B. In this model, they observed a profound reduction in expression of CXCL8 that attenuated tumor associated angiogenesis. Similarly, Xiong et al. [64], in a model system of human pancreatic carcinoma, reported that block of NF-*κ*B activity was associated with the inhibition of tumor growth, tumor-associated angiogenesis, and metastases.

The ELR+ CXC chemokines are important mediators of tumor angiogenesis and act both as autocrine growth factors and as potent paracrine mediators of angiogenesis to promote tumorigenesis and metastases. All of the angiogenic CXC chemokine promoters contain a putative* cis*-element that recognizes the NF-*κ*B family of transcriptional factors, [18, 65] suggesting that NF-*κ*B plays an important role in the transactivation of angiogenic CXC chemokines [66].

Tumor cell invasion is dependent on the ability to secrete a variety of enzymes, which include metalloproteinases (MMPs) and serine and cysteine proteinases that lead to the degradation of extracellular matrix (ECM) basement membrane and to the entry of tumor cells into the circulation.

The role of chemokines in this process is crucial; Braeuer et al. have demonstrated that CXCL8-transfected human melanoma cell lines displayed MMP-2 upregulation and increased invasiveness and metastatic potential* in vitro* and* in vivo *[67]. In addition, the transfection of human CXCL1, 2, or 3 genes into immortalized murine melanocytes led to anchorage-independent growth* in vitro* and to formation of highly vascularized tumors* in vivo* in immunodeficient mice [68, 69]. Furthermore, Coillie et al. showed that the production of CXCL6 leads to intratumoral expression of MMP9 and promotes tumor growth by increased angiogenesis in melanoma mouse model [70].

The use of CXCR4 inhibitor Plerixafor has demonstrated to impair the development of lung metastasis. In this study, murine melanoma B16 cells were injected into the tail vein of C57BL/6 CXCR4(+/+) and CXCR4(+/−) mice, reporting a significant reduction of lung metastasis in CXCR4(+/−) mice [39]. Notably, AMD11070, a novel orally bioavailable inhibitor of CXCR4, has been shown to abrogate melanoma cell migration independently from B-RAF wild-type and mutated status [40].

Similarly, overexpression of CXCL8 in prostate cancer cell lines resulted in MMP-9 upregulation and increased invasiveness [71]. The human prostate cancer cells overexpressing CXCL8 became highly tumorigenic and metastatic with associated increased angiogenesis, whereas the cells transfected with antisense CXCL8 showed reduced growth and metastatic potential [71]. Prostate cancer cell lines can utilize distinct CXC chemokines to mediate their tumorigenic potential. Serum levels of CXCL8 have been found to be markedly elevated in patients with prostate cancer and correlate with the stage of disease [72]. This observation in patients has been substantiated in human/SCID mice chimeras of human prostate cancer tumorigenesis [73]. Three human prostate cancer cell lines were examined for constitutive production of angiogenic ELR+ CXC chemokines [72]. Tumorigenesis of the human prostate cancer cell line, PC-3, was shown to be attributable, in part, to the production of CXCL8. Depletion of endogenous CXCL8 inhibited PC-3 tumor growth in SCID mice that was entirely attributable to inhibition of tumor-derived angiogenesis [73]. On the other hand, CXCL1, but not anti-CXCL8, was found to be responsible of mediating tumor-derived angiogenesis in human DU145 prostate cancer cell line [27].

Notably, G31P, a CXCR1/2 inhibitor, has been shown to inhibit prostate cancer cell growth* in vitro* and in nude mouse xenografts. Thus, G31P inhibited tumor tissue vascularization, which was associated with the decreased expression of VEGF and NF-*κ*B in orthotopic xenograft tissues [27].

As regards to CXCR4 inhibitors, Plerixafor has demonstrated to sensitize PC3-luc cells to docetaxel chemotherapy in a subcutaneous xenograft mouse model of human prostate carcinoma [28].

CXCL-8 has been determined to play a significant role in mediating human ovarian carcinoma-derived angiogenesis and tumorigenesis [23]. When human ovarian carcinoma cell lines were implanted into the peritoneum of immunocompromised mice the expression of CXCL8 was directly correlated with neovascularization and inversely correlated with survival. In the same study, the expression of VEGF correlated with ascites production; however, it was not associated either with the extent of angiogenesis or with mortality rates [24].

Scotton et al. demonstrated that CXCL12/SDF-1*α* was expressed and had multiple biological effects, including DNA synthesis and migration, in epithelial ovarian cancer [25]. In the study published by Kajiyama et al., CXCL12/SDF-1*α*/CXCR4 increased the adhesion of tumor cells onto the human peritoneal mesothelial cells (HPMCs) lining the peritoneal cavity positively [26]. Furthermore CXCR4 expression significantly predicted poorer overall survival compared with negative expression. The use of Plerixafor in nude mice inoculated with ES-2 ovarian cancer cells resulted in reduced dissemination [26].

Furthermore, non-small cell lung cancer (NSCLC) cell lines that constitutively express high levels of CXCL8 have greater angiogenic activity in mice [74, 75]. Tumor-bearing animals depleted of CXCL-8 demonstrated a 40% reduction in tumor growth and in spontaneous metastasization that directly correlated to reduced angiogenesis [76]. The direct relationship between tumor-derived CXCL-8 and tumorigenesis was demonstrated in* in vivo* model of human tumorigenesis (i.e., human NSCLC/SCID mouse chimera) [76]. In this study, when CXCL8 was depleted, there was a significant reduction in tumor size, tumor induced angiogenesis, and metastases [28].

CXCL5, as compared to CXCL8, showed a higher degree of correlation with NSCLC-derived angiogenesis [77]. A direct correlation between CXCL5 tissue levels in surgical specimens of NSCLC and the extent of capillary density consistent with tumor angiogenesis has been reported [78]. These studies were extended to a SCID mouse model of human NSCLC tumorigenesis. CXCL5 expression was directly correlated with tumor growth, tumor-derived angiogenesis, and metastatic potential. When NSCLC tumor-bearing animals were depleted of CXCL5, both tumor growth and spontaneous metastases were markedly attenuated [77]. The reduction of angiogenesis was also associated with increased tumor cell apoptosis [24].

Presently, CXCR4 is under evaluation as a potential target in NSCLC and SCLC. This notion is supported by the evidence that the activation of CXCR4 leads to lung cancer cell migration and adhesion to stromal cells, which in turn provides growth- and drug-resistance signals to the tumor cells [29].

Furthermore, TF14016, a small peptidic inhibitor CXCR4, has been recently shown to suppress metastases of SCLC cells in mice [30]. More recently, BKT140, a highly selective inverse agonist of CXCR4, was shown to reduce the colony-forming capacity of NSCLC cell lines* in vitro* and the growth of NSCLC cell line xenografts* in vivo* [31].

Glioblastoma multiforme (GBM) is characterized by high proliferation of tumor cells, increased cellularity, necrosis, and marked angiogenesis that is partly linked to hypoxia [79, 80]. Under hypoxic conditions, the hypoxia-inducible factor-1 (HIF-1) activates a large battery of genes involved in increasing O_2_ availability or in the metabolic adaptation of cells to O_2_ deprivation within their microenvironment. These genes contain hypoxic response elements (HREs) and include genes such as vascular endothelial growth factor (VEGF) [81]. Zagzag et al. reported that hypoxia regulates CXCR4 in GBMs at two levels. First, through HIF-1*α* in the pseudopalisading tumor cells and, secondly, by the VEGF-stimulated angiogenic response in human brain microvascular endothelial cells [82].

The invasiveness of GBM cells is associated with the expression of CXCL8, which is straightly connected with NF-*κ*B expression [83]. Ehtesham et al. observed that CXCR4 is highly expressed in glioma progenitor cells and its ligand CXCL12/SDF-1 promotes a specific proliferative response in these cells [42]. CXCL12/SDF-1 and its receptor CXCR4 have been shown to promote glioma stem cell-mediated VEGF production and tumour angiogenesis via PI3K/AKT signalling [43]. Currently, CXCR4 inhibitor, Plerixafor, in combination with an anti-VEGF antibody, bevacizumab, is under evaluation in a phase I study of recurrent high grade glioma patients (NCT01339039).

In addition, CXCR3 has been reported to promote tumor growth in a murine model of malignant glioma [44]. Furthermore, CXCL12/SDF-1*α* induced a significant increase of DNA synthesis in primary human glioblastoma cell cultures and chemotaxis in a glioblastoma cell line [84].

In the last decade, several studies have been focused on the use of recombinant autonomous* parvoviruses* as antitumor agents. Enderlin and colleagues reported the simultaneous use of CXCL10 and TNF-*α* vectors was able to obtain complete tumor regression in coinfected glioma cells; however, these effects were not observed in the* in vivo *model of glioblastoma [85].

The ELR+ CXC chemokines have also been studied in human gastrointestinal cancers. In colorectal cancer, tumor growth* in vivo* is associated with increased expression of CXCL1 [86]. The higher expression of CXCR4 in tumor tissue correlates with poor prognosis in colorectal cancer patients [35]. Interestingly, treatment with CXCR4 antagonist Plerixafor reduced proliferation, invasion, and extracellular signal-regulated kinase 1/2 (ERK1/2) signaling, thus sensitizing tumor cells toward cytoreductive chemotherapy in two colon cancer cell lines (HT-29 and SW480) [87]. Colorectal cancer cell adhesion to endothelium is a key event in tumor progression; CXCL12/SDF-1*α* treatment stimulates intercellular adhesion molecule-1 (ICAM-1) expression, thus promoting tumor cell adhesion [88]. Moreover, CXCR2 inhibition has been shown to profoundly suppress inflammation-driven tumorigenesis as well as spontaneous adenocarcinoma formation in a model of invasive intestinal adenocarcinoma (AhCreER; Apcfl/+; Ptenfl/fl mice) [36].

Human pancreatic cancer cell lines secrete CXCL1 and CXCL8 [89], but their expression differs across the different cell lines [90]. Furthermore, aberrant expression of CXCL16 and CXCR6 might be involved in gastric carcinogenesis [33], while CXCR4/SDF-1*α* axis may play a role in lymph node metastasis of gastric carcinoma [34].

The role of chemokines in esophageal and gastric carcinogenesis is still a matter of debate [91]. This is partially due to the fact that the role of chemokines in these settings has been only indirectly evidenced through expression/correlation studies. Jung et al. showed that levels of CXCL1 in serum samples of patients with gastric cancer were significantly higher as compared to healthy individuals. Moreover, increased CXCL1 levels correlated with advanced tumor stage and lymph node metastasis [92]. On the other hand, overexpression of CXCL1 has been shown to positively correlate with improved survival in patients with gastric adenocarcinomas [93]. Concerning CXCL8, its expression seems to be associated with tumor size, lymph node, distant metastases, and poor prognosis in patients with esophageal squamous cell carcinomas [94, 95]. Accordingly, the expression of CXCL8 correlated with tumor vascularization, aggressiveness, invasion, and metastasis and with poor prognosis in patients with gastric adenocarcinoma [96, 97].

As regards to CXCL4L1, the product of the nonallelic variant gene of CXCL4 [98], it has been shown to inhibit neovascularization and counterbalance angiogenic activity mediated by VEGF, CXCL8, and CXCL12 in esophageal and colorectal cancer [20].

Finally, CXCL6 has been shown to be expressed by endothelial cells from human patients with gastrointestinal malignancies. Its expression correlated with leukocyte infiltration into the tumor and with the MMP-9 expression, suggesting that the production of CXCL6 by endothelial cells within the tumor can contribute to endothelial cell chemotaxis and tumor cell invasion and metastasization by attracting and activating neutrophils [21].

In the study led by Hassan et al., CXCR4 was overexpressed in over 60% of breast cancer samples. Elevated expression of CXCR4 carried a poor prognosis and was strongly associated with human epidermal growth factor receptor 2 (HER2) expression in these tumors [22]. Inhibition of CXCR4 by peptide antagonist CTCE-9908 resulted in a 45% inhibition of primary tumor growth and a 42% reduction of VEGF expression levels. In combination with docetaxel or the antiangiogenic agent DC101, the reduction of primary tumor volume and distant metastasis spread were markedly enhanced, suggesting its potential use in the management of these patients [22].

In triple-negative breast cancer patients, the expression level of CXCR4 was significantly related to tumor size, advanced TNM stage, and shorter overall- and disease-free survival, while in luminal or HER2-positive breast cancer groups, CXCR4 was not correlated with clinicopathological characteristics and survival [99].

The use of Nobiletin, a citrus bioflavonoid, has been demonstrated to downregulate both of the constitutive expressions of CXCR4 and MMP-9 in human breast cancer cells. This inhibition leads to the suppression of the constitutive NF-*κ*B and MAPKs activation, thus reducing the metastatic potential of breast cancer cells [100].

CXCL12/SDF-1 via CXCR4 had been implicated in mediating angiogenesis [32, 37]. In a model of tumorigenesis and metastases of human NSCLC, Phillips et al. demonstrated that CXCR4 was predominately expressed on the tumor cells and did not mediate angiogenesis in an* in vivo* model of heterotopic or orthotopic human NSCLC [41]. The depletion of ELR− chemokine CXCL12/SDF-1*α* or its receptor CXCR4 was not associated with changes in the size of the primary tumor or in a decline in primary tumor-associated angiogenesis [76]. In contrast, a marked attenuation of tumor metastases was observed, suggesting that the CXCL12/SDF-1*α* and CXCR4 may regulate metastases in an angiogenesis-independent manner. At present, NOX-A12 is the only anticancer agent in active clinical development that specifically binds to CXCL12/SDF-1-1*α*, thereby preventing the binding of CXCL12/SDF-1*α* to its receptors CXCR4 and CXCR7 and blocking the subsequent receptor activation. NOX-A12 is under study in combination with proteasome inhibitor bortezomib and dexamethasone in relapsed multiple myeloma patients (NCT01521533) and in combination with bendamustine and anti-CD20 mAb rituximab in relapsed chronic lymphocytic leukemia (NCT01486797).

Finally, CXCR4 inhibitor Plerixafor has demonstrated to reduce the* in vitro* metastatic potential of human bladder carcinoma cell [101] and the lymph node metastases of B88 oral squamous cell carcinoma [102].

## 4. Role of ELR− CXC Chemokine in Attenuating Angiogenesis Associated with Tumorigenesis

ELR− CXC chemokines have been shown to inhibit angiogenesis in several tumor models, such as human NSCLC. Thus, in model systems of human NSCLC tumorigenesis in SCID mice inoculated with either adenocarcinoma or squamous cell carcinoma cell lines, the levels of CXCL10 were inversely correlated with tumor growth and resulted higher in squamous cell carcinoma compared with adenocarcinoma tumors [103]. The appearance of spontaneous lung metastases in SCID mice inoculated with adenocarcinoma tumors occurred after that the CXCL10 levels from either the primary tumor or plasma had reached a nadir [104]. The depletion of CXCL10 in squamous cell carcinoma tumors by the use of neutralizing anti-CXCL10 antibodies resulted in a clear increase in their size. Moreover, the administration of continuous intratumor injections of low dose, recombinant human CXCL10 (100 ng every other day) in adenocarcinoma tumors reduced both their size and metastatic potential, which was directly attributable to a reduction in tumor-associated angiogenesis [103]. Accordingly, the retroviral gene transfer of CXCL10 inhibited growth of human melanoma xenografts in an angiogenesis-dependent manner [104].

Furthermore, overexpression of CXCL9 resulted in the inhibition of NSCLC tumor growth and metastasis via a decrease in tumor-associated angiogenesis [105]. These findings support the notion that the generation of agonists to CXCR3 may represent a crucial step in the antitumor therapy.

As regards to the angiostatic role of CXCL14, in a model of human prostate cancer transfected with CXCL14, 43% reduction tumor growth as compared to controls was found [106].

Finally, in a human NSCLC tumor cell line, stable transfection or overexpression of DARC resulted in binding of angiogenic ELR+ CXC chemokines by the tumor cells. Thus, DARC acts as a decoy receptor and interferes with the ability of these angiogenic factors to stimulate endothelial cells, with a marked decrease in tumor mediated angiogenesis and metastatic potential [107]. Accordingly, in a mouse model of prostate cancer, animals with a DARC-deficient background developed larger and more aggressive tumors with greater tumor associated neovascularization and increased intratumor levels of angiogenic ELR+ CXC chemokines [108].

## 5. CC Chemokines

The CC subfamily of chemokines is defined by the arrangement of the first two of four invariant cysteine residues found in all chemokines. This large family consists of 10 CCR receptors (CCR1-10) with 25 ligands (CCL). Their main function is considered to mediate and direct the trafficking and migration of monocytes and lymphocytes. Chemokine receptors are expressed not only by circulating cells but also by tissue resident cells, including epithelia, endothelia, stromal cells, neurons, and smooth muscle, and their expression is upregulated in various inflammatory conditions [109]. Among CC chemokine family members, CCL2, CCL11, CCL16, and CCL21 have been shown to be involved in angiogenesis. CCL2-mediated angiogenesis has been demonstrated in* in vitro* [110–112] and* in vivo *[113–115] studies and seems to be directly linked to the effect of CCL2/CCR2 axis on the vascular endothelium [115]. This activity is dependent on membrane type 1-matrix metalloproteinase (MT1-MMP). Thus,* in vivo* and* in vitro* angiogenesis induced by CCL2 was decreased in the absence of MT1-MMP activity [110].* In vivo* CCL2-induced angiogenesis has been related to both inductions of VEGF-A gene expression [116] and the transcription factor, MCP-1 induced protein (MCPIP) [117].

Presently, only few studies have investigated the role of CCL2 in tumor angiogenesis [111, 115]. Stamatovic et al. observed that CCL2 played a crucial role in mediating hemangioma growth and angiogenesis [111]. Moreover, treatment of immunodeficient mice bearing human breast carcinoma cells with a neutralizing antibody to MCP-1 resulted in significant increases in survival and inhibition of the growth of lung micrometastases [115]. Recently, a human anti-CCL2 IgG1*κ* monoclonal antibody (mAb), carlumab (CNTO 888), has demonstrated to be well tolerated with evidence of transient free CCL2 suppression and preliminary antitumor activity in a phase I study of 44 patients with solid tumors [118]. On the other hand, CNTO 888 has not shown antitumor activity as a single agent in metastatic castration-resistant prostate cancer [119].

**Figure 2:**
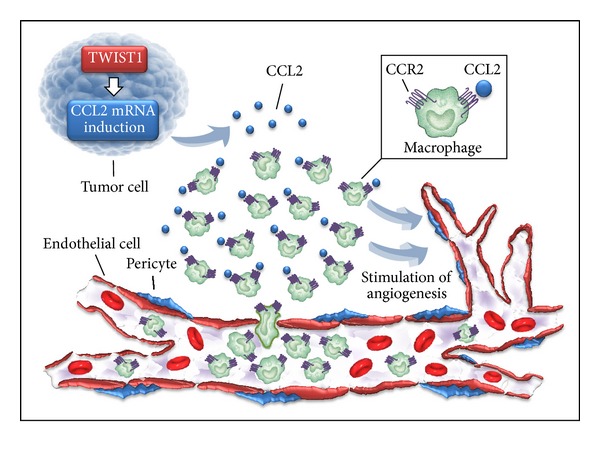
The transcription factor TWIST1 promotes angiogenesis and tumor progression without increasing the secretion of VEGF but rather by inducing the expression of the macrophage chemoattractant CCL2. Tumor cells that express Twist1 upregulate CCL2 transcript, increase CCL2 protein levels, and lead to the formation of a CCL2 gradient in tumor microenvironment. This gradient attracts macrophages, which promote tumor angiogenesis.

TWIST1 is a basic helix-loop-helix transcription factor expressed in newly formed mesenchyme cells. Low-Marchelli et al. reported that TWIST1 promoted angiogenesis and tumor progression without increasing the secretion of VEGF but rather induced expression of the macrophage chemoattractant CCL2 (Figure 2). Thus, the inhibition of endogenous TWIST1* in vivo* blocked macrophage recruitment and angiogenesis [120].

Concerning the other members of CC chemokine family, CCL11 and CCL16 have been shown to promote angiogenesis* in vitro* and* in vivo *[121, 122]. Sharma et al. have found that the CCL21 has a potent antitumor activity. In addition to a reduction in angiogenesis, intratumoral injection of a recombinant CC chemokine, CCL21, induced tumor regression in immunocompetent mice, but not immunosuppressed mice, suggesting that T cell immunity was required for the antitumor effect of CCL21 [123]. The biological effect of CCL21 was dependent on the spatial generation of intratumor IFN-*γ* and CXCR3 ligands [45, 123, 124]. Recently, CCL21 and SPARC-like protein 1 (SPARCL1/MAST9/hevin/SC-1) have demonstrated to be associated with drug resistance in ovarian cancer [125].

CCRL2 is a 7-transmembrane G protein-coupled receptor which plays a key role in lung dendritic cell trafficking to peripheral lymph nodes. CCRL2 acts as a receptor for the chemokine chemerin, also known as retinoic acid receptor responder protein 2 (RARRES2). Yin et al. reported that CCRL2 expression level is correlated with tumor grade in human glioma patient samples and cell lines. Moreover, the overexpression of CCRL2 significantly enhanced the migration rate and invasiveness of the glioblastoma cells, suggesting for a potential role of CCRL2 as a therapeutic target in these patients [126].

## 6. Conclusion 

Mounting evidence suggests that CXC and CC chemokines exhibit either angiogenic or angiostatic biological activity, which is partially due to their ability in modulating immune response. Dysregulation of this complex network can result in tumorigenesis. Nevertheless, it is commonly stated that the chemokine system is promiscuous or redundant and it is still not clear if this “functional redundancy” may carry to the impossibility of completely inhibiting single chemokine receptors. However, several findings suggest that the model of functional redundancy may be not the dominant biology of chemokine system, at least not in the case of inflammatory disease [127]. In this context, further studies are needed to achieve effective levels of chemokine antagonists by dosing them correctly.

Therefore, targeting CXC and CC chemokines and/or their receptors may provide novel opportunities for therapeutic intervention in solid tumors. Although recent findings have been very encouraging, additional studies are still necessary to test the safety and efficacy of these agents.

## Figures and Tables

**Table 1:** Expression of chemokine receptor in various solid tumors.

Cancer	Receptor expressed	References
Breast	CXCR1, CXCR2, CXCR4, and CXCR7	[7], [19], [20], [21], [22]
Ovarian	CXCR4	[7], [19], [23], [24], [25], [26]
Prostate	CXCR1, CXCR2, and CXCR4	[7], [19], [27], [28]
Lung (NSCLC)	CXCR2, CXCR4, and CCR7	[7], [19], [29], [30], [31], [32]
Esophageal	CXCR4	[7], [19]
Stomach	CXCR4, CXCR6, and CCR7	[7], [19], [33], [34]
Colorectal	CXCR2, CXCR4, CCR6, and CCR7	[7], [19], [35], [36]
Pancreas	CXCR2, CXCR3, CXCR4, and CCR7	[7], [19]
Bladder	CXCR4, CXCR7	[7], [19], [37]
Kidney	CXCR3, CXCR4, CXCR6, CXCR7, CCR2, CCR5, and CCR6	[7], [19], [38]
Melanoma	CXCR4, CXCR10, CCR7, and CCR9	[7], [19], [39], [40]
Head and neck	CXCR1, CXCR2, CXCR4, CCR7, and CXCR5	[7], [19], [41]
Brain	CXCR2, CXCR3, CXCR4, and CCRL2	[7], [19], [42], [43], [44], [45]
Osteosarcoma	CXCR4, CCR5	[7], [19]
Neuroblastoma	CXCR4, CXCR7	[7], [19]

**Table 2:** Ongoing clinical trials on chemokines and chemokine receptors in patients with solid tumors.

Trial ID number	Agent description and Study design
NCT01339039 (phase I)	AMD3100 (CXCR4 antagonist) in combination with bevacizumab in patients with recurrent high grade glioma

NCT00591682 (phase I)	MSX-122 in patients with refractory metastatic or locally advanced solid tumors

NCT01545141 (phase I/II)	IFN, celecoxib, and rintatolimod [Chemokine-modulatory (CKM) regimen] as neoadjuvant therapy in patients with recurrent resectable colorectal cancer

NCT01433172 (phase I/II)	GM.CD40L Vaccine with CCL21 in patients with metastatic adenocarcinoma of the lung who must have received and completed first line therapy

NCT01015560 (phase II)	Anti-CCR2 antibody MLN1202 in treating patients with bone metastases

NCT01736813 (phase I)	Maraviroc (CCR5 inhibitor) in previously treated colorectal cancer with liver metastasis

NCT01339975	Evaluation of the role of CXCL4, CXCL4L1 and CXCR3 as biomarkers in localized, locally advanced or metastatic renal cell carcinoma

NCT00174096	Investigation of the relationship between SDF-1/CXCR4 and metastasis of laryngeal and hypopharyngeal squamous cell carcinomas

NCT00851955	Description of the role of CXCR2 ligands/CXCR2 biological axis in patients with pancreatic cancer
